# Supplementary material for: Fatty Acid Supplementation Affects Skin Wound Healing in a Rat Model
Source: Nutrients. 2022 May 27;14(11):2245. doi: 10.3390/nu14112245 (PMC9182784; doi:10.3390/nu14112245)
Supplement: Supplementary file 1 [file nutrients-14-02245-s001.zip › nutrients-1711269-supplementary.pdf]

Supplementary file

Fatty acid supplementation affects skin wound healing in a rat model

Alica Hokynková [1,2], Marie Nováková [2], Petr Babula [2], Miroslava Sedláčková [3], Hana Paulová [4], Miroslava Hlaváčová [4], Daniela Charwátová [2], and Tibor Stračina [2,*]

[1] Department of Burns and Plastic Surgery, Faculty Hospital Brno and Faculty of Medicine, Masaryk University, Jihlavská 20, 625 00 Brno, Czech Republic
[2] Department of Physiology, Faculty of Medicine, Masaryk University, Kamenice 5, 625 00 Brno, Czech Republic
[3] Department of Histology and Embryology, Faculty of Medicine, Masaryk University, Kamenice 3, 625 00 Brno, Czech Republic
[4] Department of Biochemistry, Faculty of Medicine, Masaryk University, Kamenice 5, 625 00 Brno, Czech Republic
* Correspondence: stracina@med.muni.cz; Tel.: + 420 549 496 867

Wound closure level

The daily area that is yet to heal was measured by a noncontact method using a tablet with specialised planimetric software (Electreasure, HC Electronics, Hradec Králové, Czech Republic). The first measurement was performed immediately after surgery and then the measurements were made daily. The wound snapshot together with the calibration ruler was taken with the build-in camera (Figure S1). Each wound snapshot was calibrated according to the ruler and stored. The wound perimeters were then outlined on the display with a stylus and the area that had not yet healed (in cm^2) was calculated.

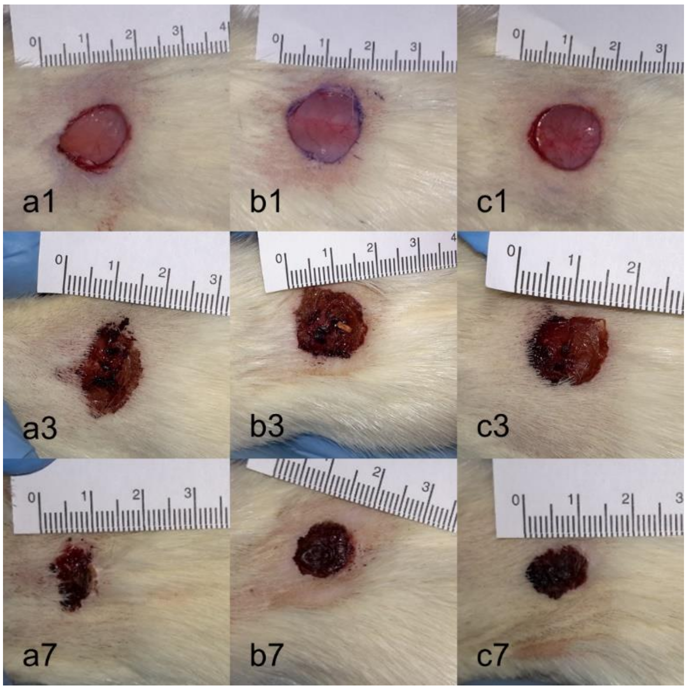

Figure S1. Representative pictures of wounds used for the planimetric analysis. Upper panels (a1-c1) show wounds on the day of surgery (day 14), middle panels (a3-c3) show wounds two day after the surgery (day 16), lower panels (a7-c7) show wounds at the day of termination (day 21); (a1, a3, a7) group A, (b1, b3, b7) group B, (c1, c3, c7) control group. The pictures in the columns represent the same animal from the group. Note the progress of wound contraction. Each picture contains the calibration ruler (numbers represent centimetres).

Hematological parameters

Basic hematological parameters – red blood cell count, hematocrit, hemoglobin concentration, red blood cell distribution width, platelets count and white blood cell count – were evaluated from EDTA blood samples. The analysis was performed immediately after sample collection using the Mythic 18 blood analyzer (Orphée SA, Switzerland). Each sample was measured twice and averaged. The results are summarized in Table S1. No significant differences were found in either of the hematological parameters.

Table S1. Hematological parameters.

**Parameter**	**Unit**	**Group A**		**Group B**		**Group C**	
		**Mean**	**S.E.M.**	**Mean**	**S.E.M.**	**Mean**	**S.E.M.**
RBC	10^{12}/l	5.72	0.13	5.53	0.10	5.67	0.11
HGB	g/l	114.15	1.92	112.22	1.82	116.50	1.84
HCT	-	0.31	0.01	0.30	0.00	0.31	0.01
MCV	fl	53.88	0.55	54.41	0.43	55.22	0.57
MCH	pg	19.99	0.15	20.33	0.19	20.58	0.21
MCHC	g/l	371.30	1.28	373.50	1.42	372.55	2.14
RDW	%	15.49	0.21	15.80	0.31	15.42	0.24
PLT	10^{9}/l	584.89	15.00	623.89	42.84	525.94	52.28
WBC	10^{9}/l	5.11	0.60	5.28	0.59	4.53	0.34
LYM	%	94.45	1.17	95.69	0.76	92.99	1.31
MON	%	3.89	0.81	3.36	0.61	3.94	0.61
GRA	%	1.24	0.34	0.96	0.18	3.07	0.95

RBC – erythrocytes; HGB – hemoglobin; HCT – hematocrit; MCV – mean corpuscular volume; MCH – mean corpuscular hemoglobin; MCHC – mean corpuscular hemoglobin concentration; RDW – erythrocytes distribution width; PLT – platelets; WBC – leukocytes; LYM – lymphocytes; MON – monocytes; GRA – granulocytes

Polyunsaturated fatty acids profile in plasma phospholipids and triacylglycerols

The tables below show polyunsaturated fatty acids (PUFAs) profiles in plasma phospholipids (Table S2) and triacylglycerols (Table S3) after two-week administration of lipid emulsion in rat. Animals in groups E and F received vegetable-derived 20% lipid emulsions (Biomedica Praha, Czech Republic) by orogastric tube once a day. Animals in control group (C) received water in corresponding volume.

Table S2. Polyunsaturated fatty acids (PUFAs) profile in plasma phospholipids. A, B – experimental groups, C – control group. Data are presented as median (lower quartile – upper quartile). White rows – n-6 PUFAs; grey rows – n-3 PUFAs.

Trivial name		A [mol %]	B [mol %]	C [mol %]
Linoleic acid (LA)	18:2n-6	21.58 (20.00; 22.55)	20.59 (19.74; 21.79)	20.21 (18.8; 22.7)
γ-Linolenic acid	18:3n-6	0.06 (0.05; 0.07)	0.06 (0.05; 0.06)	0.05 (0.03; 0.05)
Dihomolinoleic acid	20:2n-6	0.42 (0.28; 0.43)	0.44 (0.41; 0.44)	0.39 (0.32; 0.43)
Dihomo-γ-linolenic acid	20:3n-6	1.05 (0.85; 1.17)	0.92 (0.86; 1.05)	0.87 (0.74; 0.90)
Arachidonic acid	20:4n-6	19.85 (18.16; 21.18)	19.98 (19.19; 22.33)	21.23 (20.45; 21.94)
Adrenic acid	22:4n-6	0.40 (0.37; 0.47)	0.38 (0.36; 0.40)	0.43 (0.36; 0.47)
Osbond acid	22:5n-6	0.38 (0.31; 0.46)	0.37 (0.34; 0.41)	0.53 (0.47; 0.55)
α-Linolenic acid (ALA)	18:3n-3	0.11 (0.10; 0.11)	0.10 (0.09; 0.11)	0.09 (0.08; 0.11)
Timnodonic acid (EPA)	20:5n-3	0.12 (0.09; 0.14)	0.13 (0.11; 0.15)	0.04 (0.03; 0.06)
Clupanodonic acid (DPA)	22:5n-3+24:0	0.71 (0.66; 0.76)	1.02 (0.93; 1.18)	0.77 (0.63; 0.85)
Cervonic acid (DHA)	22:6n-3	3.17 (3.00; 3.56)	2.56 (2.21; 3.03)	3.11 (2.93; 3.23)
Σ n-6		43.16 (42.47; 43.83)	37.96 (35.81; 40.34)	43.50 (42.77; 44.77)
Σ n-3		4.17 (3.93; 4.45)	5.50 (4.96; 6.37)	4.02 (3.89; 4.24)

Table S3. Polyunsaturated fatty acids (PUFAs) profile in plasma triacylglycerols. A, B – experimental groups, C – control group. Data are presented as median (lower quartile – upper quartile). White rows – n-6 PUFAs; grey rows – n-3 PUFAs.

Trivial name		A [mol %]	B [mol %]	C [mol %]
Linoleic acid (LA)	18:2n-6	28.46 (27.15; 28.83)	29.51 (29.12; 30.21)	29.52 (28.25; 30.76)
γ-Linolenic acid	18:3n-6	0.30 (0.28; 0.40)	0.32 (0.30; 0.34)	0.35 (0.31; 0.39)
Dihomolinoleic acid	20:2n-6	0.56 (0.47; 0.63)	0.47 (0.45; 0.51)	0.52 (0.51; 0.56)
Dihomo-γ-linolenic acid	20:3n-6	0.53 (0.31; 0.59)	0.55 (0.46; 0.59)	0.48 (0.43; 0.51)
Arachidonic acid	20:4n-6	4.93 (3.37; 5.52)	4.70 (4.13; 6.23)	5.23 (4.58; 6.13)
Adrenic acid	22:4n-6	1.24 (0.84; 1.53)	1.20 (1.15; 1.33)	1.27 (1.20; 1.58)
Osbond acid	22:5n-6	0.79 (0.67; 1.01)	0.77 (0.68; 0.82)	1.04 (0.94; 1.12)
α-Linolenic acid (ALA)	18:3n-3	1.25 (0.99; 1.36)	1.39 (1.36; 1.48)	1.40 (1.32; 1.43)
Timnodonic acid (EPA)	20:5n-3	0.42 (0.32; 0.48)	0.51 (0.26; 0.76)	0.47 (0.36; 0.68)
Clupanodonic acid (DPA)	22:5n-3+24:0	0.93 (0.65; 1.03)	1.02 (0.93; 1.18)	0.90 (0.81; 1.07)
Cervonic acid (DHA)	22:6n-3	2.22 (1.57; 2.53)	2.56 (2.21; 3.03)	1.94 (1.76; 2.21)
Σ n-6		36.44 (34.26; 38.47)	37.96 (35.81; 40.34)	38.48 (37.12; 39.78)
Σ n-3		4.73 (3.94; 5.00)	5.50 (4.96; 6.37)	4.81 (4.42; 5.16)
